# Viscoelastic Properties in Cancer: From Cells to Spheroids

**DOI:** 10.3390/cells10071704

**Published:** 2021-07-06

**Authors:** Yara Abidine, Arianna Giannetti, Jean Revilloud, Valérie M. Laurent, Claude Verdier

**Affiliations:** Université Grenoble Alpes, CNRS, LIPhy, F-38000 Grenoble, France; yara.abidine@gmail.com (Y.A.); argianna21@gmail.com (A.G.); jean.revilloud@univ-grenoble-alpes.fr (J.R.); valerie.laurent@univ-grenoble-alpes.fr (V.M.L.)

**Keywords:** cancer cells, spheroids, AFM, confocal microscopy, rheology, microenvironment

## Abstract

AFM-based rheology methods enable the investigation of the viscoelastic properties of cancer cells. Such properties are known to be essential for cell functions, especially for malignant cells. Here, the relevance of the force modulation method was investigated to characterize the viscoelasticity of bladder cancer cells of various invasiveness on soft substrates, revealing that the rheology parameters are a signature of malignancy. Furthermore, the collagen microenvironment affects the viscoelastic moduli of cancer cell spheroids; thus, collagen serves as a powerful proxy, leading to an increase of the dynamic moduli vs. frequency, as predicted by a double power law model. Taken together, these results shed new light on how cancer cells and tissues adapt their viscoelastic properties depending on their malignancy and the microenvironment. This method could be an attractive way to control their properties in the future, based on the similarity of spheroids with in vivo tumor models.

## 1. Introduction

Cell mechanical properties are essential in various processes such as cell migration, embryogenesis, wound healing, and the immune response, i.e., physiological processes, but also in various pathological cases, in particular during cancer metastasis. Understanding how cell deformability can control the way cancer cells migrate or extravasate through blood vessels is essential. While it is well known that the elastic properties play a central role in such processes [1,2], the viscoelastic nature of the cell system is significant and has to be taken into account [3,4,5]. The many filaments (actin, microtubules, and intermediate filaments) of the cell provide a rigid structure to the cell, thus an elastic response, and the surrounding fluid embraces viscosity, providing the typical viscoelastic nature of the cell, similar to a physical gel that can remodel constantly. This is indeed what is happening as the cell migrates, and F-actin filaments polymerize at the front to push the membrane forward, while the other actin monomers are recycled [6]. Several other studies have proposed a different behavior for the cell cytoplasm, where the fluid flow can affect the mechanical response and give rise to a poroelastic behavior [7,8]. It is therefore important to obtain experimental data regarding cell behavior under various mechanical solicitations such as constant force, prescribed deformation, or rate of deformation. For this reason, new methods have been proposed to determine such properties, either locally using AFM [9,10], magnetocytometry [11], particle-tracking measurements [12], or using global measurements thanks to microfluidics [13], micropipettes [14], optical tweezers [15], or optical coherence elastography [16].

While the mechanical properties of cancer cells have been extensively studied [1,2,5], the tissue scale is less known; therefore the aim of this work was to understand how cellular properties combine and whether similar ones are retained at a higher scale. As could be expected, several features are preserved as classical tissues follow nonlinear elastic behaviors with a substantial role of the deformation rate [17]. Tissues are usually made of cells surrounded by the extra cellular matrix (ECM) inside a fluid, and the concentrations of the different components depend on tissue type. Cell suspensions have been described [18,19] using mixture models and exhibit a behavior close to that of yield stress fluids, when the cell concentration is around 50% or more. On the other end, with a large collagen content [20] and a small amount of cells (around 10%), the behavior becomes viscoelastic, but the effect of cells on the ECM is critical, since cells are able to remodel the collagen network.

To investigate other biological tissues—in particular solid tumors—spheroids have been developed over the years [21] and are considered to be outstanding in vitro models for the investigation of tumors. They are made of cells closely packed together, surrounded by the ECM in culture medium. Spheroids can grow and make their own ECM, and cell adhesion molecules (cadherins in particular) can form to bind cells within the spheroid afterwards. Several earlier studies have focused on the role of compressive stresses exerted from the outside onto the tumor [22,23], with a limiting effect on growth. Fewer results are devoted to the understanding of the mechanical behavior of such spheroids, but recent models have focused on the flow of the interstitial liquid within the spheroids [21] and revealed that poroelastic active models can fully describe them [24]. These effects have also been observed on other tissues such as cartilage or tendons, revealing notable high-frequency poroelastic responses [25,26], which can be used to differentiate physiological and pathological tissues. Although these systems are different, possible correlations can be made.

In this work, AFM was chosen as an interesting device to probe both single cancer cells and spheroids made of the same cells. A microrheology method was developed based on initial indentation followed by oscillations at various frequencies [5,27], to probe the viscoelastic properties of cells and spheroids. The method is presented in Section 3 together with the modeling tools and is used further on different cells in different environments (Section 4). Then, the model was adapted to spheroids, and similar data were obtained (Section 5); however, some differences between the two systems investigated were observed: the viscoelastic properties of both cells and spheroids depend on the microenvironment (substrate or collagen within the spheroid). Therefore, complementary confocal microscopy experiments were carried out on the spheroids to understand these behaviors, and all results are discussed in Section 6.

## 2. Materials and Methods

### 2.1. Cancer Cells

Three epithelial bladder cancer cell lines with increasing malignancy were used: RT112 (luminal molecular subtype, Leibniz Institute DSMZ, Braunschweig, Germany) and T24 and J82 (American Type Culture Collection, Manassas, VA). RT112 is a moderately differentiated cell line that exhibits low invasiveness, while T24 and J82 are both poorly differentiated and very malignant cells, with J82 being the more invasive type. The choice of such bladder cell lines came from earlier studies by the authors [28,29,30,31], and the classification method was described previously [27]. These cells were cultured in RPMI 1640 (Gibco, Saint-Aubin, France) supplemented with 10% fetal bovine serum (FBS, Life Technologies SAS, Villebon-sur-Yvette, France) and 1% penicillin-streptomycin (Life Technologies SAS). One day before the measurements, cells were seeded at a low density on polyacrylamide gels coated with 20 mg/mL fibronectin (PromoCell, Heidelberg, Germany) overnight at 37 ${}^{{}^{\circ}}$C in a humidified 5% CO${}_{2}$ atmosphere. These cells are considered to adhere firmly on such gels. AFM measurements were carried out on isolated cells at 37 ${}^{{}^{\circ}}$C. These cells were stably transfected with a plasmid expressing LifeAct-GFP to stain F-actin according to a previous work [32]. Results were collected on a reasonable number of cells in various conditions (N = 5), which can be used to extract statistical significance. More data are available in our other works [5,27].

### 2.2. Polyacrylamide Gels

Two-dimensional polyacrylamide gel substrates were prepared prior to seeding of cells using a previous protocol [27]. These gels were synthesized by mixing acrylamide (30% *w*/*w*) and N-hydroxyethyl-acrylamide (5.85% *w*/*w*) at fixed concentrations of 3.2% and 1.25%, respectively, as well as N,N-methylene-bisacrylamide (2% *w*/*w*) at three different weight concentrations (0.1–0.3–0.6%) in a 50 mM HEPES buffer. After the gels were degassed for 20 min, polymerization was initiated using 10% ammonium persulfate solution (APS) and N,N,N′,N′-tetramethylethylenediamine (TEMED). All reagents were from Sigma-Aldrich. The rigidity of the gels was modulated using the three above bisacrylamide concentrations to reach gel rigidities of 5, 8, and 28 kPa, as measured by AFM. Functionalization was then achieved using fibronectin (20 μg/mL) for one hour at room temperature. Gels were kept under humid conditions before cells were seeded.

### 2.3. Spheroids’ Preparation

Spheroids can be made of various cells inside an extracellular matrix and are reported to be good candidates for modeling tumors. This is why the same T24 cells as above were selected to prepare them. As before, T24 cells were cultured in RPMI 1640 medium supplemented with 10% FBS and 1% penicillin-streptomycin. Ninety-six-well plates were filled with a 1.5% sterile agarose solution (Abnova, Fischer Scientific, Illkirch, France), which solidified quickly at room temperature. Then, ten-thousand cells were added into the well. Finally, type I rat tail collagen (Corning, Bedford, MA, USA) at a 0.01 or 0.03 mg/mL concentration (4 ${}^{{}^{\circ}}$C and pH ∼ 7.4) was added to the medium, since collagen is a good model to mimic the microenvironment of bladder cancer cells [33]. The 96-well plate was centrifuged for 10 min at 200× *g*. Cells remained at the bottom of the well and were followed in time. After 3 days, spheroids usually presented a spherical, compact shape. Experiments were conducted then, with the spheroid diameter *D* ranging between 270 and 400 μm. Spheroids were detached with a 100 μL-pipette (chopped nozzle) and deposited into Petri dishes (TPP, to fit within the AFM setup) in culture medium. Up to three spheroids could be measured at the same time. After sedimentation and proper adhesion to the Petri dish bottom, the AFM cantilever was lowered to come into contact with the top of the spheroid, and measurements were carried out as explained below (Section 3.2). Results were collected for N = 15, 10, and 6 for the respective collagen concentrations of 0, 0.01, and 0.03 mg/mL, with spheroid sizes ranging from 300 μm to 410 μm.

### 2.4. Confocal Microscopy of Spheroids

Confocal imaging of spheroids containing 0.01 mg/mL collagen was carried out using a confocal Leica TCS SP8 (LIPhy platform). Cells with F-actin GFP labeling were imaged in the green channel (argon laser, 488 nm), and collagen was imaged using the reflectance technique in the red channel (HeNe laser, 633 nm) [20,34]. The spheroids were prepared as described earlier and allowed to sediment in a Petri dish (with a 170 μm glass coverslip bound to the bottom). Images were taken (40× Leica oil-objective) from the bottom to the median part, as it was difficult to penetrate deeper than 200 μm. Z-stacks were acquired using steps of 0.35 μm. Then, images were processed using the Fiji software.

### 2.5. Statistical Analysis

Statistical analysis was measured using a two-sample unpaired Student’s *t*-test. Statistical significance was reached for * *p* < 0.05 and ** *p* < 0.01, while *p* > 0.05 was considered nonsignificant. Means are presented with the standard error of the mean (SEM).

## 3. Microrheology Using AFM

### 3.1. Principles of Viscoelasticity

The main idea behind viscoelasticity comes from the combined properties of soft systems under applied deformations or forces. When the system is elastic, stresses (i.e., forces per unit surface, in Pa) are proportional to deformations, whereas a viscous material will flow under an applied force, with stresses proportional to the rate of deformation. In order to capture both viscous and elastic responses, one can apply a sinusoidal shear deformation $\delta \;=\;\delta_{0}\;sin\left(\omega t\right)$, where $\delta_{0}$ is the amplitude of the deformation and ω=2πf is the angular frequency (f is the frequency in Hz). At small enough deformations $\delta_{0}$, one can assume and check [5,10,35] that the shear stress response is also periodic in time $\tau \left(t\right)\;=\;\tau_{0}\;sin\left(\omega t+\phi \right)$ at the same angular frequency, where $\tau_{0}$ is the stress amplitude and where ϕ is a phase shift. Thus, one can extract the components of stress in phase (ϕ=0) and phase opposition (ϕ=π/2), i.e., the moduli G’ (elastic) and G” (viscous or loss), which appear as the coefficients in front of the sin(ωt) and cos(ωt) terms. It was found that $G^{\prime }=\tau_{0}\;cos\phi /\delta_{0}$ and $G^{\prime \prime }=\tau_{0}\;sin\phi /\delta_{0}$. The resulting complex $G^{*}=\frac{\sigma^{*}}{\delta^{*}}=G^{\prime }+iG^{\prime \prime }$ is called the complex shear modulus, and the loss angle ϕ is defined by $tan\phi =\frac{G^{\prime \prime }}{G^{\prime }}$, as the ratio of viscous to elastic stresses. Given these basic principles, let us now consider a real experiment based on the use of an AFM.

### 3.2. Oscillations Using an AFM

AFM experiments were carried out using a Nanowizard II AFM (JPK Instruments) mounted on a Zeiss microscope (Observer D1). AFM is based on the use of a tip placed on a cantilever that can be set in contact with a surface to investigate the forces due to the interactions between the tip and the substrate. The laser reflected by the cantilever onto a photodiode gives the tip displacement and therefore the force thanks to proper calibration. Calibration was performed here for the sensibility parameter (s∼ 30–80 nm/Volt), then using the thermal fluctuation method [36] to finally obtain the cantilever stiffness (k in N/m). In this study, we used soft cantilevers (MLCT, lever C, Bruker, Billerica, CA) with stiffness on the order k∼ 0.01 N/m for cells, whereas for spheroids, stiffer cantilevers (Nanosensors, TL-NCL model) were favored, with k ∼ 30 N/m. The basic idea is to perform an initial indentation $\delta_{0}$, then to superimpose small sinusoidal deformations δ at a given frequency *f*. For cells, we started from the Sneddon relationship [37] for pyramidal tips, where the applied force $F_{0}$ is related to the indentation $\delta_{0}$ by: $F_{0}=\frac{3}{4}\frac{E\;tan\theta \;\delta_{0}^{2}}{1-\nu^{2}}$, where *E* is the elastic Young’s modulus, ν is the Poisson ratio (ν∼0.5), and θ is the half-pyramid angle. This relationship is then linearized (assuming $\delta \ll \delta_{0}$), to obtain:

(1) $$G^{*}\left(\omega \right)=G^{\prime }+i\;G^{\prime \prime }=\frac{1-\nu }{3\;tan\;\theta \;\delta_{0}}\left\{\frac{F^{*}\left(\omega \right)}{\delta^{*}\left(\omega \right)}-i\;\omega b\left(0\right)\right\}$$

The final calibration regards the hydrodynamic drag created by oscillations of the cantilever in a fluid: the drag is proportional to viscosity and velocity, but also depends on geometry. Here, the geometry of the cantilever was different each time, so the best way to calibrate it was to oscillate far from the substrate (given distance *h*), then come closer to it (h ∼ 0). The drag force is a pure imaginary number $F^{*}/\delta^{*}=i\omega b\left(h\right)$, where b(h) is to be found. Using the method suggested by Alcaraz et al. [38], we found that b(0) = 6.95 × 10${}^{-6}$ N.s/m for the MLCT cantilevers that we used in a typical culture medium.

In the case of spheroids, another method was proposed using large tipless cantilevers [35] indenting the spherical aggregate. The flat cantilever indented the spheroid with initial indentations $\delta_{0}∼$ 4–8 μm, corresponding to contact radii ∼ 20–40 μm, and an area of contact ∼ 1200–5000 μm${}^{2}$. As cells were connected to each other, indentation operated over the whole spheroid, as it is known from continuum theory that a soft substrate plays a role in the measured apparent modulus [27]. The Hertz formula was now applied in a reverse manner (i.e., the rigid cantilever was in contact with the soft spheroid) and led to $F_{0}=\frac{4}{3}\frac{E\;\sqrt{R}\;\delta_{0}^{3/2}}{1-\nu^{2}}$. After linearization, one finds:

(2) $$G^{*}\left(\omega \right)=G^{\prime }+i\;G^{\prime \prime }=\frac{1-\nu }{4\;{\left(R\delta_{0}\right)}^{1/2}}\left\{\frac{F^{*}\left(\omega \right)}{\delta^{*}\left(\omega \right)}-i\;\omega b\left(0\right)\right\}$$

where *R* is the spheroid radius and the other parameters remain unchanged. In that case, the hydrodynamic drag calibration gave b(0) = 3.45 × 10${}^{-5}$ N.s/m.

Therefore, using this method, it was possible to obtain the viscoelastic data (G′,G″) over a large range of frequency, ranging from 1 Hz to 1 kHz.

### 3.3. Substrate Effects

Investigating cells in a complex environment demands evaluating the mechanical properties of the surrounding media or tissues. Typically, the values of soft tissues (excluding bones) range from 50 Pa to 20 kPa. Therefore, our studies focused on such media. When using AFM on cells plated on a soft/rigid substrate, it is important to account for its rigidity. Indeed, the cell apparent modulus is overestimated on a rigid substrate, but can be estimated for different cantilever geometries [39,40]. On the other hand, less is known about soft substrates, where the apparent modulus can be underestimated, as shown recently [27,41,42]. This is precisely what was done prior to this study. The details of the calculations can be found in our previous work [27]. Corrections made use of an equivalent medium made of the glass substrate covered by a thick gel, on top of which cells were deposited. This medium was described by a simple apparent modulus [43], and Equation (1) is modified as shown previously [27] to obtain the corrected moduli ($G^{\prime }$, $G^{\prime \prime }$).

### 3.4. Rheological Model

**Figure 1:**
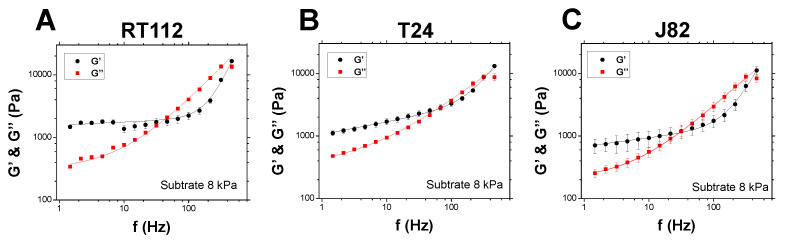
Viscoelastic moduli of RT112, T24, and J82 cells on an 8 kPa substrate. The elastic modulus $G^{\prime }$ (black) and the loss modulus $G^{\prime \prime }$ (red) are shown as a function of frequency *f*. These measurements were performed above the cell nucleus. The lines correspond to the model in Equation (3), using (**A**) $G_{0}$ = 1567 Pa, a = 0.05, $G_{1}$ = 0.005 Pa, b = 2.4, $G_{2}$ = 321 Pa, c = 0.07, $G_{3}$ = 29.4 Pa, d = 1.05, (**B**) $G_{0}$ = 1060 Pa, a = 0.20, $G_{1}$ = 0.24 Pa, b = 1.7, $G_{2}$ = 351 Pa, c = 0.08, $G_{3}$ = 84 Pa, d = 0.8 and (**C**) $G_{0}$ = 682 Pa, a = 0.14, $G_{1}$ = 0.04 Pa, b = 2, $G_{2}$ = 205 Pa, c = 0.01, $G_{3}$ = 40 Pa, d = 0.92. N = 5; error bars represent the mean ± the standard error of the mean (SEM).

Different authors have tried to model viscoelastic data. Previous studies reported that polymers or complex materials [44,45] exhibiting wide relaxation spectra have a power law dependence, i.e., moduli $G^{\prime }$ and $G^{\prime \prime }$ vary with a certain power of the frequency (f). For example, it was reported that cells have moduli ($G^{\prime }$,$G^{\prime \prime }$) varying with the same small exponents ∼ 0.1–0.3 and behave as Newtonian fluids at higher frequencies [10]. Nevertheless, a single exponent is usually not enough to cover the whole frequency range; therefore, it is better to define different frequency domains with different exponents [5,27,46]. This is what was done here. Assuming that both moduli exhibit two different behaviors according to the frequency range, as shown in Figure 1, a double power law behavior can be assumed, of the following form:

(3) $$G^{\prime }=G_{0}\;{\left(\frac{f}{f_{0}}\right)}^{a}+G_{1}\;{\left(\frac{f}{f_{0}}\right)}^{b}\;,\;\;\;\;\;\;\;\;G^{\prime \prime }=G_{2}\;{\left(\frac{f}{f_{0}}\right)}^{c}+G_{3}\;{\left(\frac{f}{f_{0}}\right)}^{d}$$

Note that we used $f_{0}=$ 1 Hz, and *f* is expressed in Hz. By making this dimensionless reduction, we can get rid of the complex units. Indeed, $G_{0},G_{1},G_{2},G_{3}$ are simply expressed in Pascals (Pa), whereas exponents a,b,c,d are dimensionless. We note further that:

- A plateau for $G^{\prime }$ is sometimes obtained at low frequencies (see Figure 3A below), so that a=0, and $G_{0}$ is the so-called elastic plateau modulus [5];
- A single power law for $G^{\prime }$ can be enough to describe the data (see Figure 3B for instance), then $G_{1}=0$;
- Note that for low frequencies, when the second term is negligible, $G^{\prime }∼G_{0}\;{\left(\frac{f}{f_{0}}\right)}^{a}$ and $G^{\prime \prime }∼G_{2}\;{\left(\frac{f}{f_{0}}\right)}^{c}$ so that $G_{0}∼G^{\prime }$ (1 Hz) and, similarly, $G_{2}∼G^{\prime \prime }$ (1 Hz);
- We also recovered most of the cases studied before; in particular, Alcaraz et al. [10] found $G_{1}=0,a=c$ and d=1 for single cells;
- In general, when two slopes are visible [46], two parameters are enough to capture the frequency dependence of the moduli (Figure 1 and Figure 3).

## 4. Results on Live Cancer Cells

### 4.1. Cell Viscoelastic Properties

The viscoelastic properties of the three different cell lines with increasing invasiveness (RT112 < T24 < J82) were measured using AFM as described in Section 3.2. The measurements were located above the cell nucleus with a maximum indentation of the AFM tip of 500 nm. Cells adhered on gels of different rigidities, and the contribution of the substrate to the viscoelastic properties was corrected as described in Section 3.3, ensuring that only the microrheology of the cells was characterized. This correction was essential as it allowed the comparison between different cell lines. Figure 1 shows the elastic modulus $G^{\prime }$ (black) and the loss modulus $G^{\prime \prime }$ (red) of cells adhering on a soft gel of 8 kPa (with stiffness similar to an endothelial monolayer [27]). First, one can observe that the viscoelastic properties followed a common behavior with $G^{\prime }$ and $G^{\prime \prime }$ increasing with frequency with the ratio of $G^{\prime \prime }$/$G^{\prime }$ when the invasiveness increased, suggesting an effect of the invasiveness on the microrheology of cancer cells. Moreover, the elastic modulus of the less invasive cells RT112 revealed a plateau modulus at the lower frequencies (Figure 1A), which was not present for T24 and J82 (Figure 1B,C), indicating that invasiveness had the effect of making cells less elastic.

**Figure 2:**
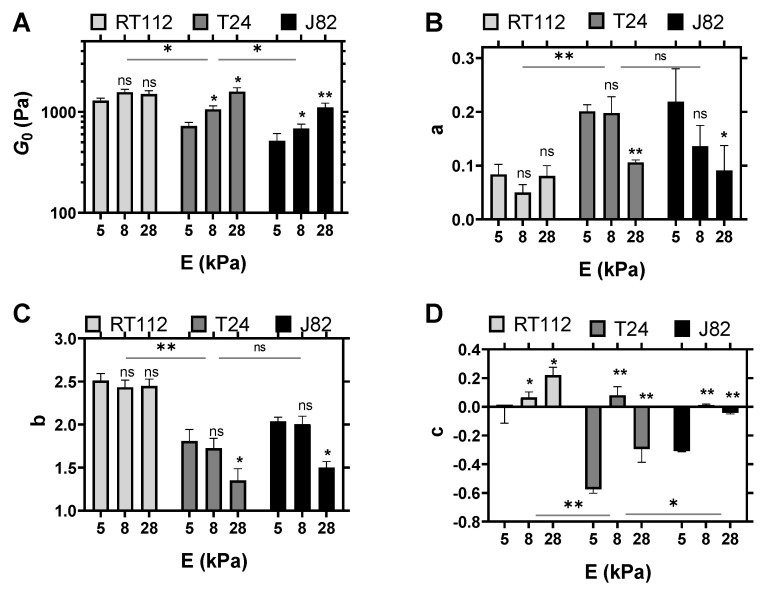
Parameters $G_{0}$ (**A**), exponent *a* (**B**), exponent *b* (**C**), and exponent *c* (**D**) for RT112, T24, and J82 cancer cells on different gels (E = 5–8–28 kPa). The parameters were extracted from fitting averaged $G^{\prime }$ and $G^{\prime \prime }$ for each condition with Equation (3). Three different adjustments were performed, and the means of the fitted parameters were obtained. N=5; error bars represent the mean ± SEM. Asterisks represent a significant difference using the Student *t*-test: ns = not significant, * *p* < 0.05 and ** *p* < 0.01. For each cell line, the statistical asterisk is versus the condition 5 kPa. A bar is added under the asterisk when a statistical difference is found between RT112–T24 and T24–J82 on 8 kPa gels.

The quantitative parameters of cancer cell microrheology were then found using the model in Equation (3) and are summarized in Figure 2 and Figure S1 (Supplementary Material). First, the elastic modulus $G_{0}$ decreased significantly between RT112 and J82 (Figure 2A), while the slope *a* of $G^{\prime }$ increased (Figure 2B). At a high frequency, $G_{1}$ of RT112 increased significantly as compared to T24 and J82 (Figure S1A), while the slope *b* of $G^{\prime }$ decreased (Figure 2C). These results indicated that the less-invasive cells were more elastic. In addition, the loss modulus $G^{\prime \prime }$ exhibited a similar dependency on the cancer cell invasiveness. Indeed, the slope *c* (Figure 2D, 8 kPa) of the more invasive cells J82 was lower than RT112 and T24 and close to zero, revealing that the loss modulus followed a single power law.

These results demonstrated that the cell microrheology parameters can provide the signature of malignancy, with more invasive cell lines being less elastic and more deformable, which gives them the advantage of rapidly changing their shape and migrating through endothelial barriers.

### 4.2. Comparison of Various Substrates

While the use of 8 kPa soft gels is biologically relevant in the context of metastasis, when cancer cells are in contact with the endothelium monolayer (having a similar rigidity [27]), it is also important to investigate the effect of the substrate rigidity on the viscoelastic properties. This is relevant to understand how cancer cells modify their microstructure depending on the substrate. Here, we used polyacrylamide gels with a calibrated stiffnesses of 5–8–28 kPa. The viscoelastic moduli were measured above the cell nucleus and corrected for the effect of the substrate, as mentioned above, and the microrheological parameters were determined using Equation (3) and are summarized in Figure 2.

These results revealed that invasive cells were sensitive to the substrate rigidity. Indeed, the rheology parameters of T24 and J82 exhibited significant changes with the gel rigidity increase. In particular, $G_{0}$ increased, while *a* and *b* (exponents at low and high frequencies, respectively) decreased when the substrate became more rigid (Figure 2A–C). This suggested that the cells became more rigid when the substrate was stiffer. Interestingly, the less invasive cell line RT112 did not show a similar behavior, indicating that the more invasive cells were more sensitive to the substrate rigidity. However, the effect of the substrate was less pronounced on the loss modulus. Indeed, the model revealed that the exponent *c* of $G^{\prime \prime }$ (low frequencies) increased significantly for RT112 and J82 with the gel rigidity (Figure 2D).

Taken together, these results revealed that the mechanosensitivity of cancer cells to the substrate depended on their invasiveness.

In summary, it was shown that the microrheology of cancer cells depended both on (i) the invasiveness of the cells and (ii) the substrate rigidity. These findings shed light on the adaptive power of the more invasive cell lines, which can deform and pass through barriers, but can also change their mechanical properties depending on the type of substrate.

## 5. Results on Spheroids

### 5.1. Spheroid Viscoelastic Properties

**Figure 3:**
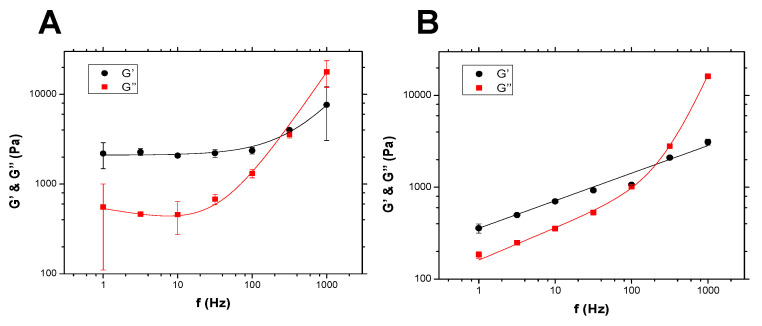
(**A**) Viscoelastic properties ($G^{\prime }$, $G^{\prime \prime }$) of a T24 spheroid with **no collagen**. D ∼320 μm. Initial number of cells = 10,000. The lines correspond to the model in Equation (3), using $G_{0}$ = 2100 Pa, a = 0, $G_{1}$ = 4.8 Pa, b = 1.02, $G_{2}$ = 530 Pa, c = −0.15, $G_{3}$ = 4.4 Pa, d = 1.2. (**B**) Viscoelastic properties ($G^{\prime }$, $G^{\prime \prime }$) of a T24 spheroid containing collagen at 0.03 mg/mL. D ∼410 μm. Initial number of cells=10,000. The same model gives $G_{0}$ = 358 Pa, a = 0.30, $G_{1}$ = 0 Pa, b = 0, $G_{2}$ = 161 Pa, c = 0.35, $G_{3}$ = 0.026 Pa, d = 1.92. Representative data are shown here with N = 2 (no collagen) and N = 4 (0.03 mg/mL); error bars represent the mean ± SEM.

Experimental data on such spheroids are interesting and already reveal features associated with cell mechanical properties, as well as spheroid content. Figure 3 shows two sets of measurements conducted on T24 spheroids: without collagen (diameter of 320 μm) in Figure 3A and with collagen at a 0.03 mg/mL concentration (diameter of 410 μm) in Figure 3B.

There was indeed a difference with respect to the low-frequency behavior with a plateau modulus for $G^{\prime }$ and $G^{\prime \prime }$ in the case without collagen (Figure 3A), whereas interestingly, when collagen was added (Figure 3B), moduli $G^{\prime }$ and $G^{\prime \prime }$ exhibited a power law behavior with no plateau at low frequencies. Modeling of this behavior was carried out using Equation (3).

### 5.2. Role of Collagen

**Figure 4:**
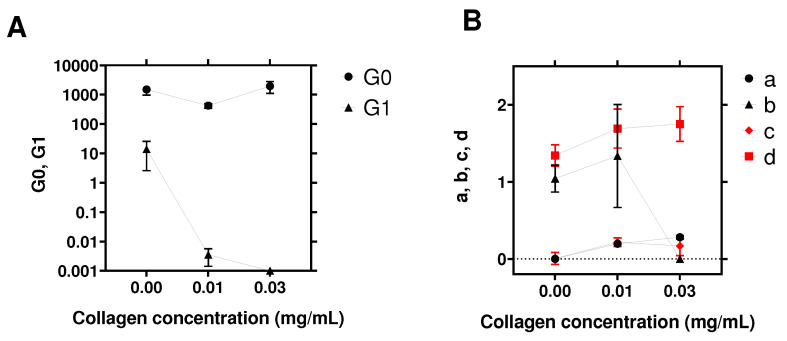
Role of collagen concentration (0–0.01–0.03 mg/mL) on parameters $G_{0}$, $G_{1}$ (**A**) and exponents a, b, c, d (**B**). The values of all parameters are reported in Table 1. For each condition, we considered three spheroid sizes ranging from 300 μm to 410 μm with $G^{\prime }$ and $G^{\prime \prime }$ averaged for each spheroid diameter (a total of N = 15, 10, 6 experiments for respective collagen concentrations of 0, 0.01, 0.03 mg/mL). The parameters were then extracted by fitting the averaged $G^{\prime }$ and $G^{\prime \prime }$ with the model in Equation (3). The error bars represent the standard error of the mean (SEM).

The role of collagen was further characterized using spheroids cultured at three different concentrations of collagen: 0–0.01 mg/mL and 0.03 mg/mL. The spheroids all had an equal number of initial cells (10,000 cells) and had diameters ranging between 300 μm and 410 μm. This ensured that the diameter would not play a role [35]. The viscoelastic properties ($G^{\prime }$, $G^{\prime \prime }$) were acquired as described previously and fitted using the model in Equation (3). The fitting parameters are summarized in Figure 4 and Table 1. First, one can observe that the exponent *a* of $G^{\prime }$ (at a low frequency) increased with the collagen concentration (Figure 4B). In particular, *a* was null when there was no collagen. Collagen also had an effect on $G^{\prime }$ at the high frequencies, where both $G_{1}$ and *b* decreased for the highest concentration of collagen (Figure 4A,B), suggesting that the spheroid modified its elastic properties when collagen was added to the microenvironment. Finally, we also show the effect of collagen on the loss modulus $G^{\prime \prime }$, where both exponents *c* and *d* (low and high frequencies) increased when collagen was present (Figure 4B).

In summary, these results demonstrated that the collagen changed the viscoelastic properties of the spheroid, in particular the low-frequency plateau modulus disappeared and gave rise to a small frequency dependence, typical of a soft glassy rheological system [45].

**Figure 5:**
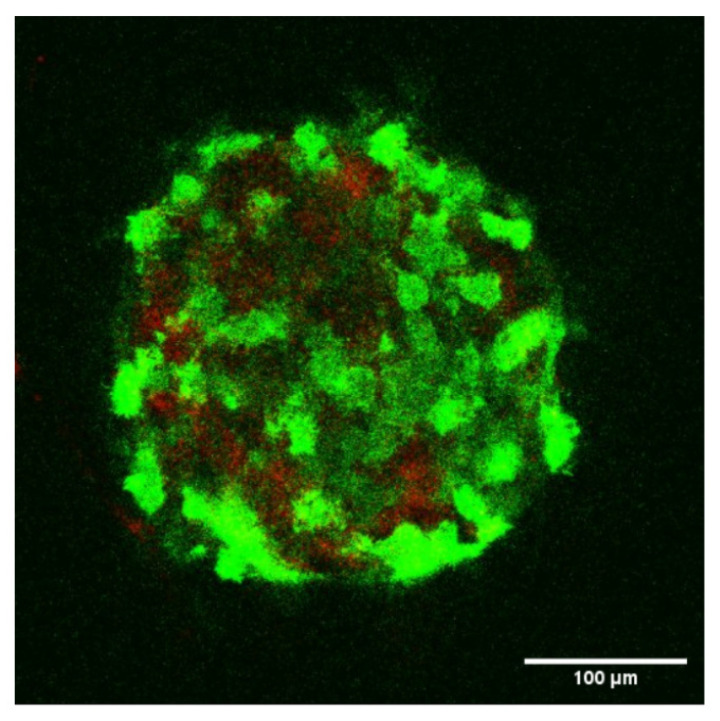
Confocal image of a T24 spheroid with 0.01 mg/mL collagen concentration (GFP cells in green; collagen in red). D ∼350 μm.

Finally, the effect of collagen on the spheroids’ microstructure was studied using confocal microscopy. This technique allowed visualizing both labeled cells (using transfection with LifeAct GFP, 488 nm) and the collagen (reflectance technique [20] at 633 nm). An example in the case of 0.01 mg/mL collagen is shown in Figure 5, where cells are visible and collagen appears in between cells. Therefore, the microstructure of the spheroid relied on the ability of cells to bind to the extracellular matrix and construct a stronger network. In the first case, there was no collagen to be seen (data not shown); however, when using collagen, this provided a network to build on, and this dramatically affected the mechanical properties, as seen above in Figure 3.

## 6. Discussion and Conclusions

The importance of the mechanical properties of cells and tissues in relation to different physiological or pathological conditions is of utmost importance [47]. Here, we proposed to determine cell and spheroid properties using dynamic rheology thanks to AFM. The technique has already been applied successfully for cells [5,10,27]. However, less is known about the rheology of tissues; in particular, only a few biological systems have been tested for viscoelasticity so far [26,48,49,50], and there were no data available for spheroids, as proposed in this new study. AFM was found to be a powerful tool for measuring elastic and loss moduli ($G^{\prime }$, $G^{\prime \prime }$) and revealed important new features. To analyze the data, a new model was used in Equation (3), based on the fact that the usual power law behaviors fail to describe single-cell or spheroid properties [27,46,51]. Indeed, it is common to use a single power law exponent describing the elastic modulus G′ in the low-frequency regime [10,52]. However, the loss modulus $G^{\prime \prime }$ has a different behavior at low and high frequencies with respective small and high slopes. This can also be the case for the storage modulus $G^{\prime }$. Therefore, the model proposed here contained two potential slopes for each modulus ($G^{\prime }$ or $G^{\prime \prime }$), allowing us to recover all possible cases (see Section 3.4); thus, the fitting curves (Figure 1 and Figure 3) approximated the data very well.

The investigation of the microrheology of single cancer cells revealed that the dynamic mechanical properties are clearly dependent on cell type (i.e., invasiveness) and substrate stiffness (Figure 1 and Figure 2). New fitting parameters using the new model suggested that possible differences between invasive or noninvasive cells can be clearly identified through the new parameters $G_{0}$ and *a*, to name just these two (see Figure 2). With respect to different substrates, cancer cells also revealed their ability to behave differently, in relation to their cytoskeleton [27,53]. The above parameters $G_{0}$ and *a*, as well as *c* and *d*, were also used successfully to exhibit this microrheology evolution when the substrate stiffness increased from 5 to 28 kPa (see Figure 2). Finally, invasive cancer cells were found to be more mechanosensitive with respect to an increase of substrate stiffness.

When treating the case of spheroids, which are known to be good tumor models [21,24,35,54], interesting data were found, regarding the role of collagen or the ECM [55]. Indeed, it is known that collagen can be synthesized by cells or can be recruited from the extracellular matrix, and in particular, collagen I was found to be a good model for the microenvironment of bladder cancer cells when studying tumorigenesis [33]. Using small amounts of collagen (here, ranging from 0 to 0.03 mg/mL), it was found that cells re-arrange efficiently in order to form a more compact structure (see Figure 5) using collagen as a proxy [24,56]. This resulted in various behaviors due to the viscoelastic nature of collagen [20,57]. Indeed, collagen has enhanced viscous properties at a low frequency and exhibits very small moduli (typically a few tens or hundreds of Pa). This effect modified quite significantly the low-frequency behavior of the spheroids, as shown by comparing Figure 3A,B. Initial slopes of ∼ 0.3–0.35 are found for $G^{\prime }$ and $G^{\prime \prime }$ in Figure 3B, and this was quite different from the case without collagen (Figure 3A). In the presence of collagen, the spheroid behavior became that of a glassy system, probably because cells rearranged constantly in a disordered way to try to find a stable configuration, which could occur if adhesion proteins are expressed and enhance the elastic effects at longer times (as compared to the ones used in this study). Finally, the analysis of rheological parameters (Figure 4) showed that $G_{0}$ and *a* are quite relevant, as with single cells, but we further noticed that $G_{1}$, *b*, *c*, and *d* can also predict such changes. Altogether, these parameters may be quite essential for investigating the microstructural evolution of spheroids in time, when considering the main interactive components such as cells, ECM, fluid, and adhesion proteins [20].

The final question relies on the ability to describe a macroscopic system (i.e., tissue level) with a constitutive equation taking into account all the components. Here, assuming that cells within the spheroid usually look more rounded, the comparison can be made with cell measurements on the nucleus. Obviously, the signature of single cells with ($G^{\prime }$, $G^{\prime \prime }$) varying as power law models is persistent at the macroscopic level (spheroid), and this could be a starting point for future modeling. This is an interesting question raised by the homogenization of poroelastic media containing inclusions/cells embedded in a matrix [58]. Such studies could be very useful since both microscopic and macroscopic properties have been measured in this study. Additionally, the active behavior of cells [24] could be considered. However, this requires further investigations.

Therefore, these results confirm the interest in AFM rheology to investigate cells and tissues; it provides mechanical parameters (or cues) to test biological samples in the time or frequency domains, possibly leading to therapeutic investigations.

## Figures and Tables

**Table 1:** Values of the fitting parameters of $G_{0},a,G_{1},b,G_{2},c,G_{3},d$ for different collagen concentrations (0, 0.01 and 0.03 mg/mL).

Parameters	c = 0	c = 0.01 mg/mL	c = 0.03 mg/mL
$G_{0}$ (Pa)	1492	423	1946
*a*	0	0.2	0.3
$G_{1}$ (Pa)	14	0.004	0
*b*	1	1.3	0
$G_{2}$ (Pa)	364	197	1152
*c*	0.0005	0.22	0.17
$G_{3}$ (Pa)	3.6	0.6	2.8
*d*	1.34	1.69	1.75

## Data Availability

Data are available from the authors upon request.

## Supplementary Materials

The following are available online at https://www.mdpi.com/article/10.3390/cells10071704/s1, Figure S1: Parameters G1 (A), G2 (B), G3 (C) and exponent d (D) for RT112, T24 and J82 cancer cells on different gels (E = 5–8–28 kPa).
