# Supplementary material for: Viscoelastic Properties in Cancer: From Cells to Spheroids
Source: Cells. 2021 Jul 6;10(7):1704. doi: 10.3390/cells10071704 (PMC8304080; doi:10.3390/cells10071704)
Supplement: Supplementary file 1 [file cells-10-01704-s001.zip › cells-1268511-supplementary.pdf]

Supplementary materials

Viscoelastic properties of cancer systems: from cells to spheroids

Yara Abidine, Arianna Giannetti, Jean Revilloud, Valerie M. Laurent and Claude Verdier

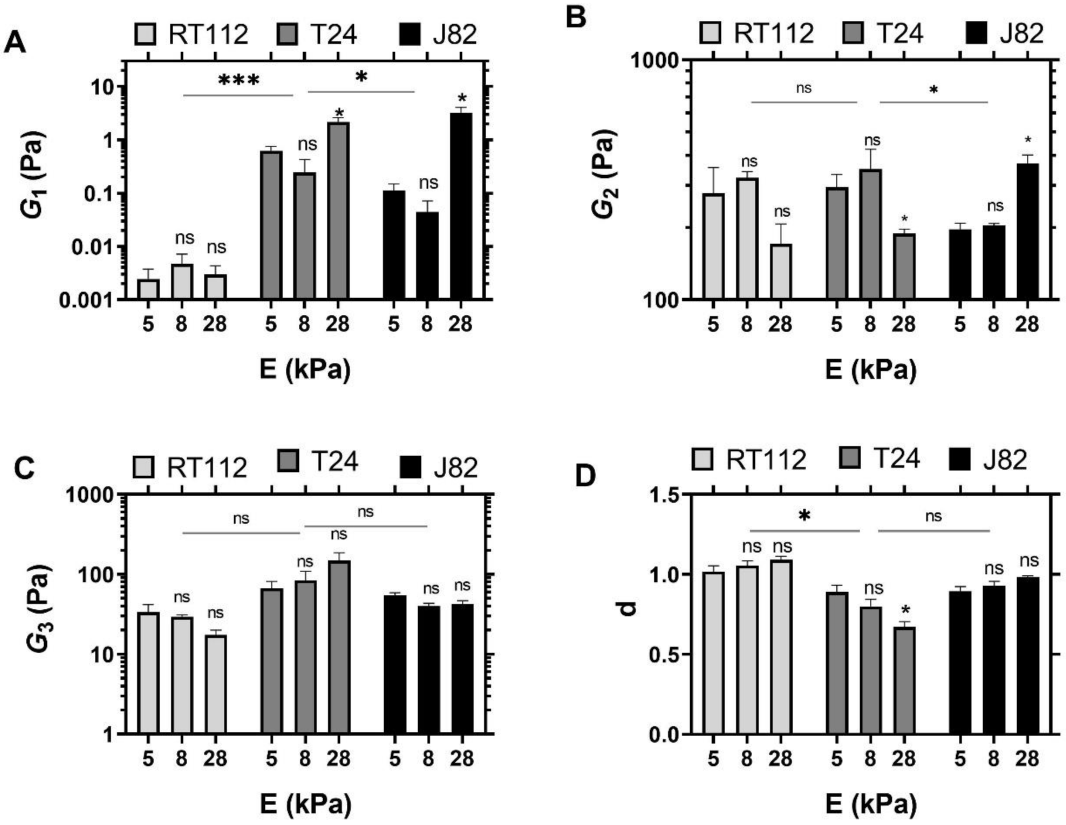

Figure S1. Parameters G_1 (A), G_2 (B), G_3 (C) and exponent d (D) for RT112, T24 and J82 cancer cells on different gels (E= 5-8-28 kPa). These parameters are a complement of the fitted parameters shown in Figure 2. The parameters were extracted from fitting G' and G'' with Eq. (3). Three different adjustments were performed, and the means of the parameters were obtained. Error bars represent the mean and SEM (standard error of the mean). Asterisks denote a significant difference by student t-test: ns=not significant, *$p<0.05$, **$p<0.01$ relative to condition 5 kPa. A bar is added under the asterisk when a difference is found between RT112-T24 and T24-J82 on 8 kPa gels.
